# Transforming Wheat Straw into Superabsorbent Polymers for Sustainable Agricultural Management

**DOI:** 10.3390/gels11120953

**Published:** 2025-11-27

**Authors:** Andrey V. Sorokin, Aidar I. Kadyirov, Igor A. Saranov, Egor M. Tsimmer, Vladislav A. Kiselev, Ivan A. Zhuravlev, Maria S. Lavlinskaya

**Affiliations:** 1 Polymer Science and Colloid Chemistry Department, Voronezh State University, 1 Universitetskaya Square, 394018 Voronezh, Russia; 2 Biophysics and Biotechnology Department, Voronezh State University, 1 Universitetskaya Square, 394018 Voronezh, Russia; 3 Institute of Power Engineering and Advanced Technologies, FRC Kazan Scientific Center of the RAS, 2/31 Lobachevsky Street, 420111 Kazan, Russia; 4 Research Core Center “Testing Center” of Voronezh State University of Engineering Technologies, Voronezh State University of Engineering Technologies, 19 Revolutsii Avenue, 394036 Voronezh, Russia

**Keywords:** wheat straw processing, superabsorbents, swelling, nutrient retention, mechanical strength of superabsorbents

## Abstract

The massive accumulation of agricultural waste, such as wheat straw, and its disposal by burning pose significant environmental challenges. This study explores a sustainable solution by converting wheat straw into composite superabsorbent polymers (SAPs)—superabsorbents contain both synthetic and biodegradable fragments—for improved agricultural water and nutrient management. Wheat straw (WS) was sequentially processed via acid and alkaline hydrolysis to yield fractions with different lignin contents, which were then carboxymethylated (CMWS-Ac and CMWS-Al) to enhance hydrophilicity. These derivatives were incorporated at 20 and 33 wt. %. into SAPs synthesized by copolymerization with acrylamide and acrylic acid. The CMWS-Al-based SAPs exhibited superior properties, including higher equilibrium swelling ratios (up to 566 g/g in water), excellent mechanical strength, and robust gel structure, as confirmed by rheological studies. Furthermore, SAPs demonstrated a significant capacity to retain urea in sand columns, with SAP-CMWS-Al-33 achieving 56% urea retention, highlighting their potential for mitigating fertilizer leaching. The results establish a correlation between the extent of straw processing, the physicochemical properties and lignin content of the derivatives, and the performance of the final SAPs. These wheat straw-based SAPs present a promising, sustainable technology for enhancing soil moisture retention, improving fertilizer use efficiency, and valorizing agricultural waste.

## 1. Introduction

Global climate change poses significant challenges to all spheres of human activity. For instance, land desertification leads to a substantial decline in the economic efficiency and crop yields of agriculture. A common response to such agricultural challenges is to expand cultivated area through deforestation or to increase fertilizer application. This extensive agricultural approach creates numerous environmental issues, including soil erosion and degradation due to the removal of protective forest belts and excessive agrochemical use; and the accumulation of unused plant biomass from expanding cultivation, which is typically burned, exacerbating global warming [1,2].

Consequently, finding a balance between preserving the global climate and ecological systems on one hand and maintaining agricultural economic efficiency on the other is a critically important and relevant issue for researchers across scientific disciplines. A promising strategy involves implementing innovative technologies and intensifying closed-loop crop production systems that utilize raw materials and feedstocks derived from agricultural waste [3]. A pertinent example is the Russian Federation, where annual wheat production exceeds 80 million tons, resulting in vast accumulations of waste, primarily straw. This straw lacks practical application [4,5] and is often burned, leading to greenhouse gas emissions.

From a compositional standpoint, wheat straw is a valuable source of polysaccharides (up to 90%), predominantly cellulose [6], representing a valuable raw material with broad potential for chemical technologies. Given the significant water-adsorbing capacity of polysaccharides [7], a promising avenue is to incorporate agricultural waste into water-absorbing materials, such as composite superabsorbents. Numerous superabsorbents (SAPs) with high water-absorption capacity have already been developed from plant-based polysaccharides, including starch [8,9,10,11], cellulose and its derivatives [12,13,14,15,16,17,18,19,20], pectins [21,22,23,24], and various gums [25,26,27,28,29,30].

Among various waste valorization strategies, SAP production offers a dual advantage: the conversion of unused waste into products that enhance agricultural efficiency and optimize the use of agrochemicals. The application of superabsorbents in agriculture significantly enhances efficiency by reducing irrigation water consumption by up to 35%, increasing crop yields by up to 80%, and optimizing the use and bioavailability of agrochemicals via a slow-release mechanism [31]. Furthermore, their use protects soil from water and wind erosion, thereby preserving fertility. Incorporating agricultural waste into these SAPs improves their eco-friendliness by introducing biodegradable properties and reduces synthesis costs by lowering the demand for commercial reagents [32]. Therefore, utilizing agricultural waste to synthesize superabsorbents for agricultural use could optimize crop processing and establish waste-free cycles within the sector.

Another challenge in agriculture that can be addressed using SAPs is the excessive application of nitrogen fertilizers. Commonly used nitrogen compounds are highly soluble and are easily leached from the soil, leading to soil acidification and contamination of the hydrosphere. In contrast, the use of hydrophilic SAPs helps retain these compounds within the target root zone [33]. This approach not only reduces costs for the agro-industry but also exerts a positive ecological impact.

Due to its biological origin, wheat straw (WS) has a variable composition that depends on its source and growth conditions. Consequently, using WS to synthesize superabsorbents yields inconsistent results, even under identical synthesis conditions. For instance, the equilibrium swelling ratio of the final products can vary by nearly an order of magnitude [34,35]. This variability hinders the scaling of these processes for industrial production.

From the other hand, straw, like any lignocellulosic material, has a complex composite structure comprising components of diverse nature—primarily polysaccharides (cellulose and hemicelluloses) and lignin, a polymer of phenylpropane links known for its high compressive strength. Consequently, by varying the depth of processing, different fractions with distinct properties (e.g., mechanical strength, water absorption capacity) can be isolated from wheat straw. Utilizing these separate fractions in superabsorbent synthesis allows for the creation of materials with tailored characteristics, significantly broadening their potential applications in agricultural management.

The aforementioned factors necessitate a systematic study of the influence of wheat straw composition and its derivatives on the physicochemical properties of the synthesized superabsorbents at the laboratory scale.

**Scheme 1:**
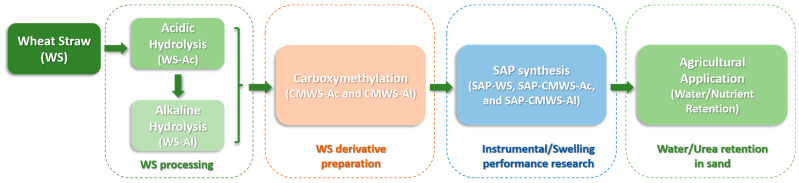
Overall experimental design flowchart.

Therefore, the aim of this work is to synthesize composite superabsorbents from various products derived from processed wheat straw, to investigate their physicochemical properties, and to evaluate their potential as agents for retaining nitrogen fertilizers in soil. To the best of our knowledge, this work presents the first comprehensive study on the utilization of variously modified wheat straw products for superabsorbent synthesis, establishing a critical link between their structural-functional characteristics (e.g., residual lignin content) and rheological properties and swelling performance of the resulting polymers, thereby demonstrating their potential for agricultural technologies. The overall experimental design is outlined in Scheme 1.

## 2. Results and Discussion

### 2.1. Wheat Straw Processing: Hydrolysis and Carboxymethylation

**Figure 1:**
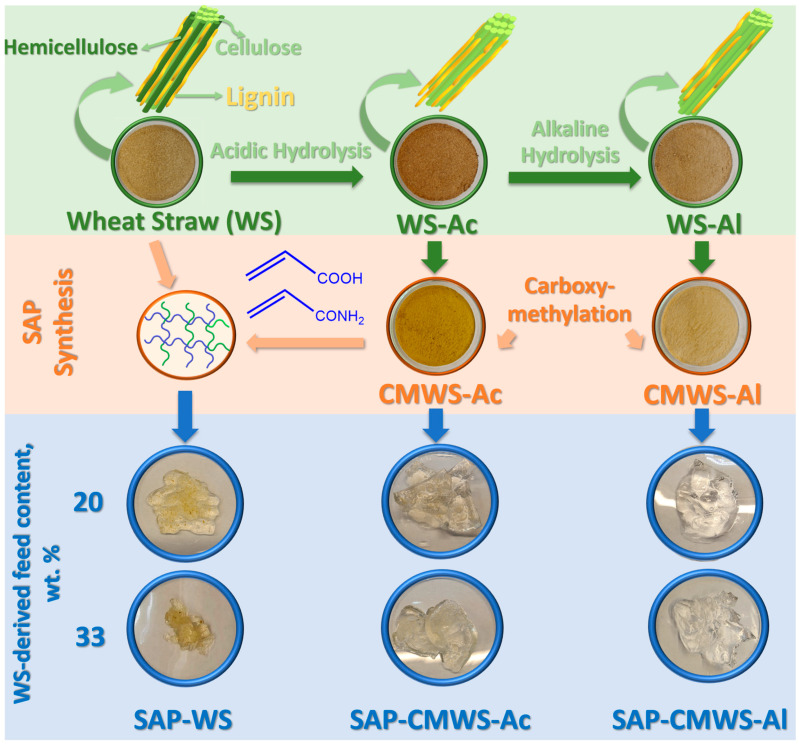
Experimental design for the synthesis of SAPs from wheat straw. Colors denote key stages: green (WS processing), peach (WS modification), and blue (SAP synthesis).

Straw is a complex biocomposite, primarily comprising cellulose microfibrils embedded in a hemicellulose matrix and reinforced by a lignin binder (Figure 1, green panel). Each of these components possesses valuable physicochemical and mechanical properties, making them attractive for the production of superabsorbents. Furthermore, their specific combination allows for the fine-tuning of the final material’s characteristics. Therefore, the initial stage of this research focused on the processing of wheat straw (WS), involving sequential acid hydrolysis (WS-Ac) and alkaline hydrolysis (WS-Al).

**Figure 2:**
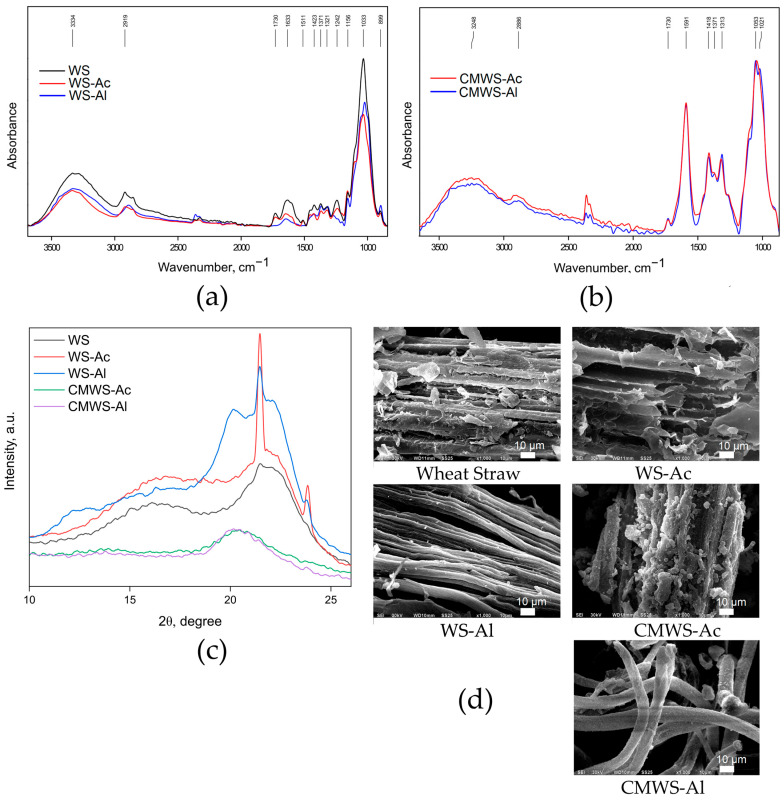
Characterization of wheat straw and its modified derivatives: (**a**) FTIR spectra of hydrolysis products, (**b**) FTIR spectra of carboxymethylated products, (**c**) XRD patterns, and (**d**) SEM images.

The FTIR spectrum of raw wheat straw (Figure 2a) exhibits characteristic absorption bands of its polysaccharide components (cellulose and hemicellulose). These include a band at 1033 cm^−1^ (C–O stretching and pyranose ring vibration), 1156 cm^−1^ (C–O–C glycosidic linkage stretching), and multiple bands between 1321–1423 cm^−1^, alongside a band at 2919 cm^−1^, assigned to C–H bending and stretching vibrations, respectively. Additional bands at 1633 and 1730 cm^−1^ are attributed to C=O stretching, while a broad band centered at 3334 cm^−1^ corresponds to O–H stretching [36]. The spectrum also reveals signals indicative of lignin, featuring a band at 1242 cm^−1^ (C–O stretching in phenols) and another at 1511 cm^−1^ (C=C stretching of aromatic rings) [37].

The FTIR spectrum of WS-Ac reveals a decrease in the intensity of bands associated with polysaccharides (e.g., at 1033, 1633, 1730, and 3334 cm^−1^, and in the 1321–1423 cm^−1^ region), whereas the band characteristics of lignin remain virtually unchanged. This indicates that the acid hydrolysis step primarily degrades the readily hydrolysable hemicelluloses, leaving the lignin content largely unaffected. In contrast, the spectrum of the subsequent alkaline hydrolysis product (WS-Al) shows a recovery in the intensity of the polysaccharide bands and a significant reduction in the lignin-associated signals. This demonstrates the effective degradation of the lignin framework by alkaline hydrolysis. This conclusion is corroborated by quantitative data from the Klason lignin method, which determined the lignin content to be 24 ± 3 wt. %. in WS-Ac and 5 ± 1 wt. % in WS-Al.

Both WS-Ac and WS-Al are insoluble in water and exhibit limited swelling, precluding their direct use in the aqueous synthesis of superabsorbents. To enhance their hydrophilicity, the products were subsequently carboxymethylated (Figure 1, peach panel). Carboxymethylation of native wheat straw yielded a product with a low degree of substitution (DS < 0.3). This is presumably because the readily soluble hemicelluloses, which are the primary components susceptible to alkylation, are partially degraded and washed out during the alkaline reaction and subsequent purification.

Figure 2b presents the FTIR spectra of the carboxymethylated products: CMWS-Ac, derived from the acid-hydrolyzed straw, and CMWS-Al, derived from the sequentially hydrolyzed (acid and alkaline) straw. The spectra retain the characteristic polysaccharide absorption bands described previously and also display new bands at 1313, 1418, and 1591 cm^−1^. These are assigned to the C–H bending of the carboxymethyl group and the symmetric and asymmetric stretching vibrations of the carboxylate ion, respectively [38]. The characteristic lignin bands are not discernible in the CMWS-Ac spectrum, as they are obscured by the more intense signals from the carboxymethyl groups.

Furthermore, the intensity of these carboxymethyl-related bands is notably higher for CMWS-Al, consistent with its higher degree of substitution (DS = 0.67 ± 0.10) compared to that of CMWS-Ac (DS = 0.45 ± 0.08), despite identical modification conditions. The lower degree of substitution for CMWS-Ac is likely due to the presence of residual lignin in its structure, which sterically hinders the accessibility of the polysaccharides for carboxymethylation.

CMWS-Al forms homogeneous aqueous solutions at concentrations of at least 1 wt. %., which are virtually free of visually detectable impurities. In contrast, solutions of CMWS-Ac contain a small quantity of water-insoluble particles, attributable to its residual lignin content.

The crystalline structure of wheat straw is significantly altered by different processing methods. Figure 2c shows the X-ray diffraction (XRD) patterns of raw wheat straw and its modified derivatives. The diffractogram of raw straw displays the two characteristic peaks of cellulose I at approximately 2θ = 16.1° and 21.5°, corresponding to the (110) and (002) lattice planes, respectively [39]. The crystallinity index (CrI) of WS is 25.23.

The XRD pattern of WS-Ac reveals a new signal at 2θ = 20°, characteristic of the (110) plane of cellulose II [39]. A concurrent increase in the intensity of the peak at 2θ = 21.4° suggests a higher relative content of cellulose I compared to the native straw. A faint reflection with diminishing intensity is also observed at 23.8°, which is typical for a semicrystalline polymer. WS-Ac exhibits a higher CrI value of 46.03 compared to WS (25.23), which is attributed to the removal of poorly organized hemicellulose.

Subsequent alkaline hydrolysis reduces the intensity of these faint reflections and the peak at 2θ = 21.4°, while concurrently intensifying the signal at 2θ = 20°. This indicates a partial degradation of the cellulose I allomorph and a concomitant increase in the cellulose II content. This suggestion is supported by the crystallinity index (CrI) value, which is 43.90 for WS-Al and lower than that of WS-Ac (46.03).

The final carboxymethylation step induces significant structural amorphization in both hydrolysis products. The XRD patterns of CMWS-Ac and CMWS-Al show a loss of distinct crystalline peaks, yielding only a broad halo centered at 2θ ~ 20°. This halo corresponds to residual, disordered fragments of the cellulose crystalline structure. Notably, the overall diffraction intensity is lower for CMWS-Al (CrI = 17.71) than for CMWS-Ac (CrI = 20.50), which is attributed to its higher degree of substitution. The introduction of more carboxymethyl groups leads to a more extensive disruption of the crystalline lattice.

The structural changes in wheat straw and its modified derivatives were visualized using scanning electron microscopy (SEM; Figure 2d). Raw WS exhibits a composite structure with a distinct layered organization, characteristic of its cellulose, hemicellulose, and lignin components. Acid hydrolysis (WS-Ac) disrupts this integrity, degrading the hemicellulose sheath and exposing underlying cellulose fibrils. This exposure is further pronounced in the WS-Al sample, where the fibrils appear fully liberated. Subsequent carboxymethylation transforms the surfaces of both CMWS-Ac and CMWS-Al, yielding characteristic “fluffy” morphologies that are typical of carboxymethylated products and confirm the successful modification.

### 2.2. Superabsorbent Synthesis and Instrumental Characterization

Wheat straw and its constituent components represent valuable feedstocks for superabsorbent polymer production. Consequently, raw straw and its modified derivatives, CMWS-Ac and CMWS-Al, were used to synthesize composite SAPs. The polymers were synthesized via radical precipitation copolymerization with acrylamide, 70% mol. neutralized acrylic acid, and the crosslinker *N*,*N*′-methylene-*bis*-acrylamide (Figure 1, peach panel). The monomer ratios, chosen based on previous work [17,40,41] to maximize the equilibrium swelling ratio, were kept constant. The biomass (WS and its derivatives) was incorporated at 20 wt. %. (SAP-20 series: SAP-WS-20, SAP-CMWS-Ac-20, SAP-CMWS-Al-20) and 33 wt. %. (SAP-33 series: SAP-WS-33, SAP-CMWS-Ac-33, SAP-CMWS-Al-33). Higher biomass loadings hindered the polymerization, leading to heterogeneous products.

The synthesized SAPs exhibit distinct visual characteristics (Figure 1, blue panel). Both SAP-CMWS-Al-20 and SAP-CMWS-Al-33, derived from the readily soluble CMWS-Al, form homogeneous, transparent gels. In contrast, SAP-CMWS-Ac-20 and SAP-CMWS-Ac-33 contain minor heterogeneities visible to the naked eye; the abundance of these inclusions increases with the CMWS-Ac content in the SAP formulation. The most pronounced particulate content is observed in the SAP-WS series, with SAP-WS-33 containing a greater quantity of visible particles than SAP-WS-20.

**Figure 3:**
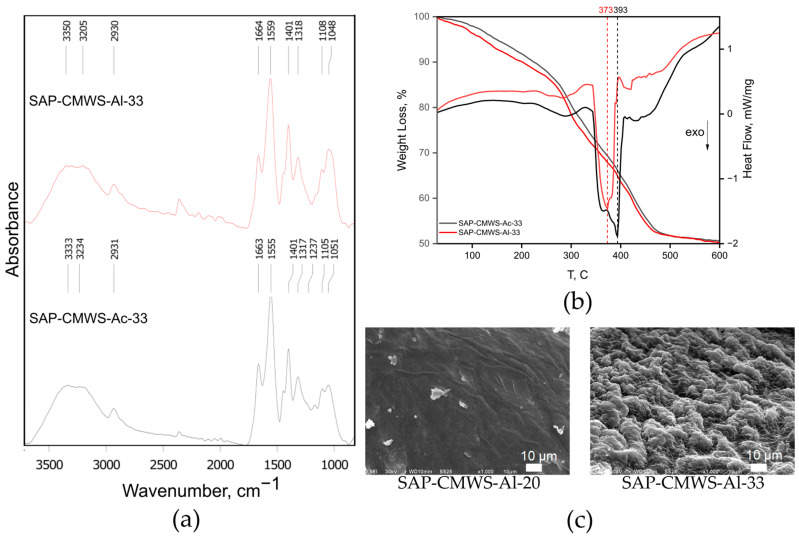
Characterization of the synthesized SAPs: (**a**) FTIR spectra, (**b**) TGA and DSC curves, and (**c**) SEM images.

The structure of the synthesized SAPs was confirmed by FTIR spectroscopy. Since the spectra of all samples are typical for such materials, Figure 3a displays the representative FTIR spectra of SAP-CMWS-Al-33 and SAP-CMWS-Ac-33 for clarity. The spectra confirm the presence of key functional groups: polysaccharides from the wheat straw derivatives (bands at ~1048–1108 cm^−1^); dissociated carboxylate groups from acrylic acid residues and carboxymethyl fragments (1317, 1401, 1555 cm^−1^ for SAP-CMWS-Ac-33; 1318, 1401, 1559 cm^−1^ for SAP-CMWS-Al-33); amide groups from acrylamide and *N*,*N*’-methylene-*bis*-acrylamide (Amide I, 1663 and 1664 cm^−1^). The Amide II band overlaps with the asymmetric carboxylate stretch near 1555 cm^−1^ [40]; methylene groups (~2931–2930 cm^−1^); and associated hydroxyl and amino groups, indicated by broad bands in the 3200–3400 cm^−1^ region [41]. Unlike the spectrum of SAP-CMWS-Al-33, the spectrum of SAP-CMWS-Ac-33 exhibits a band at 1237 cm^−1^, which is attributed to vibrations from residual lignin.

The thermal behavior of the synthesized SAPs was investigated over a temperature range of 28 to 600 °C. Both the SAP-20 and SAP-33 series exhibited similar thermal degradation behavior. The thermogravimetric analysis (TGA) results for SAP-CMWS-Ac-33 and SAP-CMWS-Al-33 are presented in Figure 3b. These samples were selected to evaluate the effect of residual lignin on the thermal stability of the synthesized SAPs. The TGA profiles reveal three distinct degradation stages. The first stage (28–300 °C) corresponds to the loss of absorbed water and dehydration of carboxyl groups on the grafted chains, with mass losses of approximately 20% and 22% for SAP-CMWS-Ac-33 and SAP-CMWS-Al-33, respectively. The second stage (~300–460 °C) involves the rapid pyrolysis of polyacrylate fragments [42], accounting for ~46% and ~47% mass loss for SAP-CMWS-Ac-33 and SAP-CMWS-Al-33. This pyrolysis is accompanied by intense exothermic peaks in the DSC profiles, with maxima at 373 °C for SAP-CMWS-Al-33 and 393 °C for SAP-CMWS-Ac-33. The final stage (~460–600 °C) shows a mass loss of ~50% for both SAPs, resulting from the decomposition of the modified wheat straw biomass. Consequently, the cumulative mass loss for SAP-CMWS-Ac-33 in the first two stages (T < 460 °C) is 1–2% lower than that of SAP-CMWS-Al-33. This difference is presumably due to the presence of residual lignin in SAP-CMWS-Ac-33, which undergoes primary decomposition at temperatures below 250 °C [43]. This interpretation is corroborated by the similar residual mass of ~50% for both samples at 600 °C, by which point lignin decomposition is complete.

So, the thermal analyses demonstrate that the synthesized SAPs are thermally stable below 100 °C, as mass losses in this range are attributable solely to the evaporation of absorbed water.

The morphology of the superabsorbents is significantly influenced by the biomass content incorporated during synthesis. This morphology—whether smooth, porous, or heterogeneous—directly governs the material’s sorption properties and, consequently, its agricultural performance. The surface of SAP-CMWS-Al-20 exhibits slight topographic variations (Figure 3c). This non-smooth morphology, which contrasts with the smooth surface typical of polyacrylates, is common for SAPs with a low polysaccharide content [41]. In contrast, SAP-CMWS-Al-33 displays a more developed surface with distinct “fluffy” formations characteristic of carboxymethylated polysaccharides (Figure 2d). As the following sections will demonstrate, these morphological differences significantly influence the sorption behavior of the superabsorbents.

Mechanical strength and rheological properties are critical parameters determining the suitability of SAPs for agricultural use. For example, readily disintegrating SAPs can function as binding agents to cement particles in light soils, thereby mitigating erosion. Conversely, robust gels with mechanical strength comparable to soil particles can enhance soil remediation and aeration. To evaluate these properties, rheological studies were conducted on the synthesized SAPs. The samples were tested at a twenty-fold swelling degree to simulate their hydrated state in soil.

**Figure 4:**
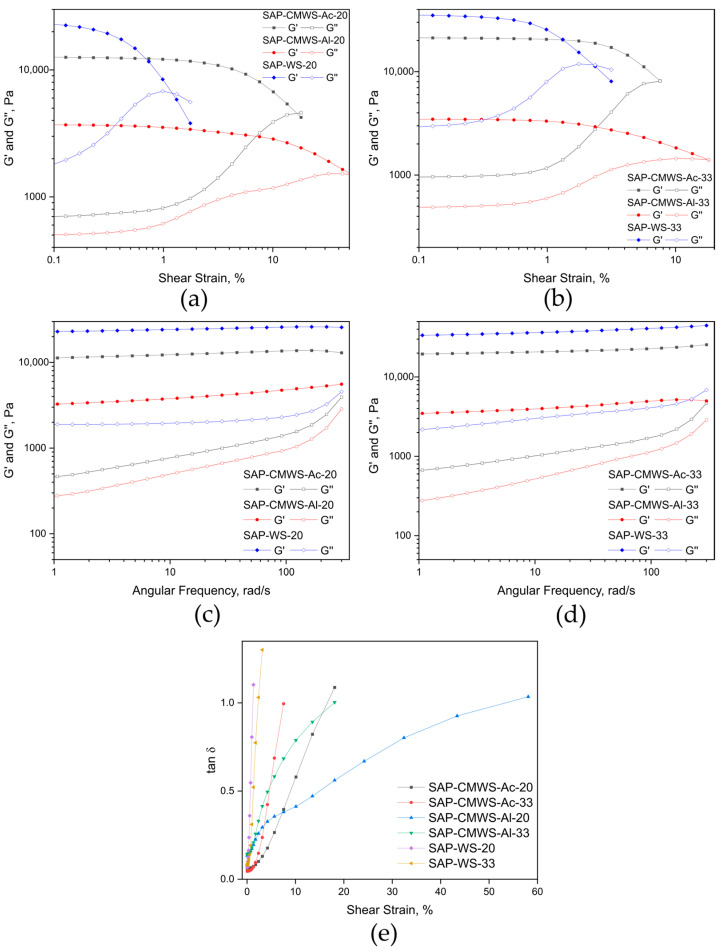
Storage (G′) and loss (G″) moduli of the SAPs as functions of shear strain (**a**,**b**) and angular frequency (**c**,**d**). Mechanical loss tangent (tan δ) of the SAPs versus shear strain (**e**).

Figure 4a,b present the storage (G′) and loss (G″) moduli as a function of strain amplitude for SAPs containing 20 and 33 wt. %. straw biomass, respectively. All samples maintain the relationship G′ > G″ across a broad deformation range, confirming their gel-like or soft solid behavior. This indicates that the material structures are sufficiently robust for elastic properties to dominate over viscous dissipation.

Within the SAP-20 series, SAP-CMWS-Ac-20 exhibits the highest storage modulus, surpassing both SAP-WS-20 and SAP-CMWS-Al-20. The SAP-WS-20 sample shows closely spaced G′ and G″ values and a narrow linear viscoelastic (LVE) region, indicating network instability even at small deformations (<1%). This confirms that unmodified wheat straw, being a complex biocomposite, integrates poorly into the polymerization, thereby acting as an essentially inert filler that participates minimally in forming the chemical network. Samples SAP-CMWS-Ac-20 and SAP-CMWS-Al-20 exhibit a LVE region than SAP-WS-20, with the most extensive region observed for SAP-CMWS-Al-20. This is likely due to the greater availability of functional groups in CMWS-Al for participation in the polymerization reaction, resulting in a more robust and elastic polymer network. Conversely, the absolute values of G′ and G″ are higher for SAP-CMWS-Ac-20 than for SAP-CMWS-Al-20. This may be attributed to the role of residual lignin, whose presence is evident in the FTIR spectrum (Figure 3a), in forming a mechanically stronger, more densely cross-linked polymer structure. These mechanical findings are consistent with the swelling properties (will be discussed further) of the SAPs: the “softer” SAP-CMWS-Al-20 swells more rapidly and to a greater extent than the “stiffer” SAP-CMWS-Ac-20.

The same trends are observed for the SAP-33 series; however, the absolute values of the storage and loss moduli are higher across all samples. The behavior of SAP-WS-33 is particularly notable, as the high straw content appears to form a percolating cellulose framework that provides initial structural stability but is readily disrupted under minor deformation.

For effective application as soil amendments, SAP’s elastic modulus should exceed that of the soil, which typically ranges from 10^3^ to 10^6^ Pa [44,45]. Therefore, SAP-CMWS-Ac-20 and all SAP-33 samples meet this fundamental criterion and demonstrate high potential for use in soil remediation and aeration.

Figure 4c,d show the results of the frequency sweep tests, displaying G′ and G″ as a function of angular frequency at a small strain within the LVE region. The persistence of G′ > G″ across the entire frequency range for all samples confirms the formation of stable, structured gels. The relatively flat, frequency-independent profiles of the moduli further corroborate their solid-like character. For the SAP-WS-20 sample, the close proximity of the G′ and G″ curves, particularly at low frequencies, indicates a weak network susceptible to slow structural rearrangements (creep) under a constant load. In contrast, the more pronounced separation between G′ and G″ for SAP-CMWS-Ac-20 and SAP-CMWS-Al-20 signifies more dominant elastic behavior and greater structural integrity.

A qualitative enhancement in mechanical properties is observed across all SAP-33 samples, which aligns with the results from the strain amplitude tests. Unlike SAP-WS-20, the SAP-WS-33 sample forms a fully developed gel with good elastic properties, although its G′ remains lower than that of the other superabsorbents. The G′ and G″ profiles for SAP-CMWS-Ac-33 and SAP-CMWS-Al-33 are nearly parallel and exhibit minimal frequency dependence, indicating a stable, robust network structure.

The frequency-dependent viscoelastic behavior reveals the long-term stability of SAPs under constant soil pressure. While most SAP-20 samples show low G′ at low frequencies, indicating susceptibility to creep, the SAP-33 series and SAP-CMWS-Al-20 maintain high, stable G′. This ensures structural integrity under sustained load, preserving soil pore space for long-term agricultural efficacy.

In the final stage of the rheological analysis, the mechanical loss tangent (tan δ = G″/G′) was analyzed as a function of strain amplitude (Figure 4e). This parameter quantifies the ratio of viscous to elastic dissipation: if tan δ < 1, elastic properties dominate (gel-like or solid-like behavior). A lower tan δ corresponds to a more elastic and mechanically robust material. If tan δ > 1, viscous properties dominate (liquid-like behavior). An increase in tan δ with strain indicates the onset of structural yielding.

Rheological analysis ranks the SAPs by mechanical strength as follows: SAP-CMWS-Al-33 > SAP-CMWS-Al-20 > SAP-CMWS-Ac-33 > SAP-CMWS-Ac-20 > SAP-WS-33 > SAP-WS-20. Most samples undergo strain softening beyond ~10–20% deformation, marked by a rising tan δ. The most robust gels (SAP-CMWS-Al-33, SAP-CMWS-Al-20) maintain the lowest tan δ, indicating superior structural integrity.

A lower tan δ directly correlates with greater stiffness, predicting superior resistance to soil compression and better shape recovery—key traits for agricultural amendments. Consequently, SAP-CMWS-Al-33 (tan δ < 0.2), SAP-CMWS-Al-20, and SAP-CMWS-Ac-33 (tan δ~0.2–0.25) are the most promising candidates for moisture-retention and soil aeration. The remaining SAPs are better suited for erosion control.

### 2.3. Superabsorbent Swelling Performance

The liquid absorption capacity is a critical performance parameter for SAPs, defining their potential applications. Commercial SAPs employed in Russian agriculture, which have proven effective for enhancing crop yields and optimizing irrigation, typically exhibit swelling degrees of 400–500 g/g (Table 1). Consequently, these values serve as a relevant benchmark for developing and evaluating new superabsorbent materials.

The equilibrium swelling ratios (ESRs) of the synthesized superabsorbents are summarized in Table 1. A clear inverse correlation is observed between the swelling capacity and the plant biomass content: higher filler loading results in lower water uptake. This trend is attributed to the inherent properties of wheat straw polysaccharides, whose macromolecular chains are densely packed into fibrils stabilized by extensive hydrogen bonding. This configuration sterically hinders a significant fraction of the hydroxyl groups, rendering them inaccessible for water sorption. This phenomenon, where a rigid biopolymer framework restricts swelling, is consistent with previous reports for superabsorbents based on chitosan derivatives [41] and carboxymethyl cellulose [40].

The type of biomass filler significantly influences the ESR, yielding the following trend: SAP-WS < SAP-CMWS-Ac < SAP-CMWS-Al. Native wheat straw is a lignocellulosic biocomposite where cellulose microfibrils are shielded by a hydrophobic lignin sheath containing fewer water-binding functional groups compared to polysaccharides. Furthermore, the presence of residual hydrophobic lignin reduces the ESR values for the SAPs based CMWS-Ac. Sequential hydrolysis and carboxymethylation partially remove the lignin barrier and disrupt the native crystalline structure. This is evidenced by XRD analysis, which shows that CMWS-Ac is more crystalline than CMWS-Al, and by SEM, which reveals that the modified cellulose fibrils are available for interaction with comonomers and water (Figure 2c,d). Collectively, these modifications enhance the accessibility and availability of hydrophilic sites. Furthermore, CMWS-Al has a higher degree of substitution than CMWS-Ac. This also explains the higher ESR values observed for these SAPs: hydrogen bonds formed between water molecules and the dissociated, charged carboxylate groups are more energetically favorable than those with the unionized hydroxyl groups of cellulose.

The swelling of ionic hydrogels and SAPs is primarily driven by electrostatic repulsion between like-charged moieties within the polymer network [46]. Since CMWS-Al has a higher degree of carboxymethylation than CMWS-Ac, the resulting SAP-CMWS-Al exhibits a greater degree of ionization. This enhanced charge density leads to stronger electrostatic repulsion, which consequently increases the swelling capacity.

This mechanism is further corroborated by the equilibrium swelling (*Q_e_*) measurements in a 0.15 M sodium chloride solution (Table 1). The saline environment suppresses polyelectrolyte dissociation, leading to a sharp decrease in swelling for all samples. This effect is most pronounced for SAP-CMWS-Al, consistent with its greater reliance on electrostatic repulsion for swelling.

Notably, the superabsorbents derived from CMWS-Al and CMWS-Ac exhibit ESRs that are competitive with, or superior to, those of commercially available analogs such as Aquasorb^®^ and Aquasin^®^ (Table 1). From the other hand, Ma et al. synthesized superabsorbents from wheat straw treated with an ammonia and nitrate, ESRs in distilled water ranging from 43.48 to 133.76 g/g [34]. Separately, Liang et al. produced wheat straw-*g*-poly(acrylic acid) superabsorbent composites with ESR values in distilled water between 498 and 860 g/g [35]. Consequently, the swelling performance of the SAPs obtained in this work is competitive with these reported benchmarks.

**Figure 5:**
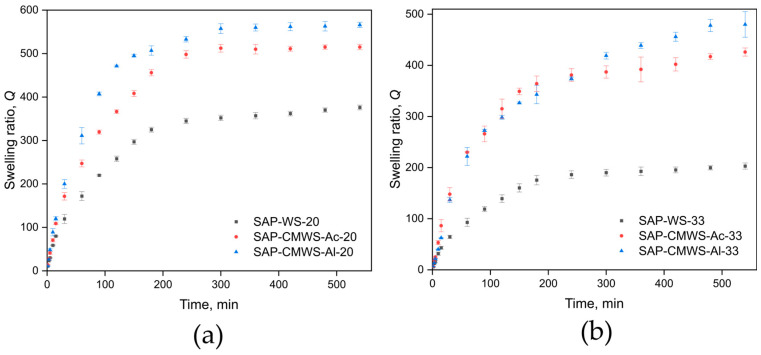
Swelling kinetic profiles in distilled water for SAP-20 series (**a**) and SAP-33 series (**b**).

To elucidate the hydration dynamics, the swelling kinetics of all samples were characterized in distilled water (Figure 5). The profiles are characteristic of ionic superabsorbents, exhibiting distinct regions of rapid initial uptake followed by a gradual approach to equilibrium, which corresponds to the saturation of the hydrogel network.

The experimental swelling data were fitted with established kinetic models commonly used for superabsorbent hydration. The applicability of each model was evaluated based on the coefficient of determination (R^2^), with the pseudo-second-order model providing the best fit for all SAPs (Table 2, Figures S1 and S2). Unlike the pseudo-first-order model, which attributes the swelling rate predominantly to surface hydration, the pseudo-second-order model accounts for hydration occurring both on the particle surface and within its bulk. This mechanism involves water diffusion into the superabsorbent matrix followed by its subsequent “fixation” via hydrogen bonding with functional groups—primarily hydroxyl, carboxylate, and amide moieties within the polymer network. Consequently, these specific interactions govern the overall hydration kinetics of the superabsorbent.

The mechanism and rate of water penetration—defined by the diffusion type—also govern the swelling behavior. To analyze this, data were fitted to the Ritger–Peppas model, with the diffusional exponent *n* determined graphically. This model is most accurate during the early to intermediate stages of swelling (up to 60% of the equilibrium swelling ratio). For all superabsorbents, the diffusional exponent *n* falls within the range 0.5 < *n* < 1 (Table 2), indicating non-Fickian (anomalous) diffusion. In this regime, the rate of water ingress is comparable to the relaxation rate of the polymer network [47]. This behavior is consistent with the experimental conditions (25 °C), under which the superabsorbents are in a glassy state, given their glass transition temperature of approximately 190–200 °C [40,47].

Analysis of the diffusion coefficient (*D*) reveals an increasing trend in the order: SAP-WS < SAP-CMWS-Ac < SAP-CMWS-Al. This progression correlates with the equilibrium swelling ratios and results from the increasingly open composite structure of the straw achieved through more extensive processing. A notable secondary trend shows that for each biomass type, the *D* value is consistently higher in the SAP-20 series than in the corresponding SAP-33 sample. Since these coefficients were determined during the rapid swelling phase (<60% of ESR), we hypothesize that surface morphology is a determining factor. The SAP-33 series features a more developed surface enriched with amorphous CMWS-Ac and CMWS-Al fragments, as clearly shown by SEM data (Figure 3c). This intricate morphology likely facilitates enhanced water penetration and mobility within the amorphized superabsorbent matrix.

Thus, by varying the type and amount of biomass used in the synthesis, superabsorbent polymers with different swelling ratios can be produced, allowing them to be tailored for specific conditions. For instance, the removal of extreme moisture (e.g., from flooding or prolonged rainfall) would favor the use of the SAP-33 series, while the SAP-20 series would be more suitable for less demanding, milder conditions. Furthermore, the swelling performance of most synthesized SAPs is competitive with commercial analogs.

### 2.4. Urea Retention in Soil

The intensification of modern agriculture is heavily reliant on the application of significant quantities of fertilizers, particularly those containing nitrogen. Urea, with the highest nitrogen content (46%) of all solid fertilizers, is consequently used extensively due to its economic efficiency. However, its high solubility facilitates rapid leaching from soil. Runoff from fertilized land can transport urea into aquatic systems, including rivers, lakes, and coastal waters [48]. Within these environments, microbial processes convert urea to ammonium and subsequently nitrate. This nutrient enrichment promotes accelerated algal growth, and the subsequent decomposition of this algal biomass consumes dissolved oxygen, leading to hypoxic “dead zones” that are detrimental to aquatic life. Therefore, developing strategies to retain urea within the plant root zone is crucial for enhancing nutrient-use efficiency, improving economic returns, and mitigating environmental damage.

Table 3 summarizes the urea retention results for sand-superabsorbent polymer mixtures. In the control experiment using pure quartz sand, only a minimal volume of the urea solution was retained upon percolation, and the effluent urea concentration remained largely unchanged. The sand itself retained only 13.9% of the initial urea.

Incorporating the SAP-20 series into the sand significantly increased the volume of urea solution held within the column. While the retained volumes for SAP-CMWS-Al-20 and SAP-CMWS-Ac-20 were comparable, the effluent from the SAP-CMWS-Al-20 mixture exhibited a substantially lower urea concentration. This suggests that the enhanced cellulose accessibility and content in SAP-CMWS-Al-20 facilitate more extensive hydrogen bonding with urea molecules within the polymer network. Consequently, the urea retention capacities for the SAP-CMWS-Al-20 and SAP-CMWS-Ac-20 mixtures were 35.7% and 30.8%, respectively.

The SAP-33 series demonstrated a further increase in the retained solution volume, likely due to the higher polysaccharide content and greater specific surface area of these SAPs. Notably, the urea concentration in the effluent for this series was only slightly lower than that for SAP-CMWS-Al-20. Ultimately, the SAP-CMWS-Ac-33 mixture retained 39.4% of the urea, while the SAP-CMWS-Al-33 mixture achieved a maximum retention of 56%. This superior performance is consistent with the highest water diffusion coefficient previously measured for this sample.

In conclusion, the SAP-33 series, and particularly SAP-CMWS-Al-33, is highly effective at retaining urea, showing significant potential for use in anti-leaching materials and slow-release fertilizer formulations.

## 3. Conclusions

This study presents an integrated approach for converting an agricultural by-product—wheat straw—into high-performance superabsorbent polymers with tailored properties for sustainable agricultural management. Sequential acid and alkaline processing, followed by carboxymethylation, produced modified derivatives (CMWS-Ac and CMWS-Al) with varying lignin content and degree of substitution. This compositional variation is a key determinant of the final materials’ characteristics. The composite SAPs, particularly those derived from the fully hydrolyzed and carboxymethylated product (CMWS-Al), exhibited competitive and superior properties. These included a high-water absorption capacity (up to 566 g/g), rivaling or exceeding commercial analogs, and enhanced rheological characteristics—such as a high storage modulus and low mechanical loss tangent—indicating an ability to maintain structural integrity under soil pressure and provide effective aeration. Furthermore, they demonstrated efficient urea retention in a model system, reaching 56% for SAP-CMWS-Al-33, highlighting their potential for smart slow-release nitrogen fertilizer systems. However, these promising results have so far been demonstrated only at the laboratory scale. Although the proposed method for converting wheat straw into functional materials employs processes common in the chemical industry, scaling this specific approach remains a significant challenge and requires further experimentation and analysis.

Our further works will focus on investigating the influence of lignin on the biodegradation of SAPs in soil. The high content of phenolic fragments in lignin enables the development of SAP-based systems for the controlled release of various agrochemicals, such as plant hormones (e.g., auxins, gibberellins, and cytokinins). Carboxymethylated lignin shares a structural similarity with auxin hormones and may act as a plant growth stimulant [49]. Systematic studies on the effect of the developed compositions on the growth and productivity of agricultural crops will help to better assess the economic and environmental advantages of creating a closed-loop system. Such a system aims to improve soil health, conserve water, and reduce pollution by converting waste into valuable products.

Consequently, developed SAPs represent an environmentally and economically sustainable solution that addresses several critical challenges in modern agriculture simultaneously: efficient water use, optimized fertilizer application, waste valorization, and climate change adaptation.

## 4. Materials and Methods

### 4.1. Materials

Wheat straw was obtained from a farm in the Voronezh region, washed with distilled water, dried at 40 °C to a constant weight, and milled to a particle size of less than 1 mm using a laboratory mill prior to use. Sodium hydroxide and sulfuric acid (both chemical grade), obtained from Vekton, Saint Petersburg, Russia, was used in the wheat straw hydrolysis. Acrylamide (AAm; extra pure, >98%), acrylic acid (AA; extra pure, >98%), *N*,*N*′-methylene-*bis*-acrylamide (MBAAm; >98%), potassium persulfate (PPS; >98%), and potassium hydroxide (>98%), all provided by Acros Organics, Geel, Antwerpen, Belgium, were used in the SAP synthesis. Urea (analytical grade, Vekton, Saint-Petersburg, Russia) was used in retention experiments. Distilled water (18.2 MΩ·cm, pH 6.7 ± 0.2), methanol, propanol-2, ethanol, acetone (all analytical grade), and ethyl acetate (chromatography grade, >99%), both provided by ReaKhim, Moscow, Russia, were used as solvents.

Prior to use, AA was distilled under vacuum (boiling point = 45 °C/15 mmHg) and was subjected to 70% mol. neutralization using potassium hydroxide. AAm and MBAAm were recrystallized from ethyl acetate, and PPS was recrystallized from deionized water. Other chemicals were used without any treatment.

Commercial superabsorbent polymers available in the Russian Federation were used as reference samples: Akvasin^®^ (LLC “Trading House ‘Singer’”, Tatarstan, Russia; https://www.tdsinger.ru/?ysclid=mi1m6jm24b682817337, accessed on 16 November 2025) and Aquasorb^®^ (SNF Group, Rhône-Alpes, France; https://snf-agriculture.com/product/aquasorb/, accessed on 16 November 2025).

### 4.2. Wheat Straw Processing

#### 4.2.1. Wheat Straw Acidic Hydrolysis

A 10 g sample of milled wheat straw was placed in a round-bottom flask equipped with a reflux condenser under atmospheric conditions. In total, 100 mL of a 1% (*w*/*w*) sulfuric acid solution was added to the flask. The resulting mixture was refluxed for 30 min. Upon completion, the precipitate was collected by vacuum filtration and dried at 60 °C to a constant weight. The product yield, denoted as WS-Ac, was 73%.

#### 4.2.2. Wheat Straw Alkaline Hydrolysis

The dried, acid-hydrolyzed wheat straw was placed in a round-bottom flask equipped with a reflux condenser under atmospheric conditions. A total of 100 mL of a 17.5% (*w*/*w*) sodium hydroxide solution was added. The mixture was refluxed for 2.5 h. Subsequently, the precipitate was isolated by vacuum filtration and dried at 60 °C to a constant weight. The product yield, denoted as WS-Al, was 36% of the initial wheat straw weight.

#### 4.2.3. Carboxymethylation

A 1 g sample of the raw material (WS, WS-Ac, or WS-Al) and 6.75 mL of a 20% (*w*/*w*) aqueous NaOH solution were placed in an Erlenmeyer flask and stirred continuously at room temperature for 1 h under atmospheric conditions. Separately, 4.66 g of sodium chloroacetate was dispersed in 20 mL of ethanol. The resulting dispersion was added to the flask and stirred for 10 min. To ensure the complete dissolution of sodium chloroacetate, the resulting mixture was placed in an ice bath and sonicated for 15 min using an UZD 2-0.1/22 ultrasonic disperser (22 kHz, 0.1 kV; Vologdin All-Russian Scientific Research Institute of High-Frequency Currents, St. Petersburg, Russia). The resulting suspension was then left under constant stirring and room temperature overnight. Finally, the precipitate was isolated, washed three times with 50 mL of ethanol without any pH adjustment, and dried at 60 °C to constant weight. The product yield is 130–157% relative to the wight of the initial raw material.

The degrees of carboxymethylation, *DS*, were determined by back-titration according to the method described in [50]. In brief, The H-form of the Na-derivatives was prepared by treating the 10 g of the sample with 30 mL of 6 M HCl for 30 min with stirring. The product was filtered, washed with methanol-water (80/20 *w*/*w*) until filtrate conductivity reached ~25 μS/cm, re-dispersed in acetone, filtered, and vacuum-dried at 50 °C.

For *DS* determination, ~0.5 g of the sample was dissolved in 20 mL of 0.2 M NaOH and diluted to 100 mL. After dilution, 25 mL aliquot was titrated with standardized 0.05 M HCl and phenolphthalein indicator. The *DS* was calculated using Equation (1) from the mean HCl volume (triplicate measurements, blank-corrected):

(1) $$DS=\;\frac{162\times \;n_{COOH}}{m_{ds}-\;{58\times n}_{COOH}},$$

where 162 and 58 are the molar masses of the anhydroglucose unit and the carboxymethyl group mass increment, g/mol, respectively; *m_ds_* is the dry sample mass, *g*, and *n_COOH_* is the molar quantity of carboxyl groups calculated as following:

(2) $$n_{COOH}=\left(V_{b}-\;V\right)\times C_{HCl}\times 4,$$

where *V* and *V_b_* are the *HCl* titrant volumes for the sample and blank, L, respectively; *C_HCl_* is the *HCl* concentration, mol/L; and the factor 4 accounts for the dilution from the 25 mL aliquot to the total 100 mL solution volume.

The determined *DS* values were 0.45 ± 0.08 for CMWS-Ac, and 0.67 ± 0.10 for CMWS-Al.

#### 4.2.4. Lignin Content Assay

The lignin content in WS-Ac and WS-Al was determined by the Klason lignin method, as described elsewhere [51]. In brief, a precisely weighed ~1 g of the biomass, previously dried to constant mass, was dispersed in 15 mL of a 6:1 (*v*/*v*) mixture of 75% H_2_SO_4_ and concentrated H_3_PO_4_. The mixture was maintained at 60 °C for 20 min. Subsequently, 185 mL of distilled water was added, and the solution was boiled for 3 h. After cooling to room temperature, the solution was vacuum-filtered through an ashless filter paper. The filter paper containing the residue was dried in an oven at 50 °C to constant mass. The dried residue and filter were then asked in a muffle furnace at 500 °C to constant mass. The lignin mass was calculated as the mass difference between the dried residue and the resulting ash.

### 4.3. Superabsorbent Synthesis

For the synthesis of SAPs, varying quantities of WS, or Ac-WS or Al-WS (see Table 4) were dissolved in 7.5 mL of distilled water under vigorous stirring in a thermostated reactor. The reactor was equipped with a condenser, a mechanical stirrer, and a nitrogen inlet for degassing. Subsequently, 2.5 mL of an aqueous solution containing AAm, AA, MBAAm, and PPS (Table 4) was introduced into the reactor. The reaction mixture was maintained at 80 °C for 2 h

The resulting product was ground, soaked in ethanol overnight to extract soluble impurities, and vacuum-dried to constant weight. The product yield after drying was 88–96%. The final SAPs were found to be insoluble in water, ethanol, isopropanol, and acetone.

### 4.4. Instrumental Characterization

#### 4.4.1. Fourier-Transform Infrared Spectroscopy

The chemical structures of both the modified wheat straw and the synthesized SAPs were analyzed by Fourier-transform infrared (FTIR) spectroscopy in attenuated total reflectance (ATR) mode. Analysis was conducted on a Bruker Vertex 70 spectrometer (Bruker Corporation, Billerica, MA, USA) fitted with a single-reflection diamond ATR accessory. Spectra were acquired across a wavenumber range of 850 to 4000 cm^−1^, accumulating 32 scans over 4 repeated cycles for each powdered sample.

#### 4.4.2. Scanning Electron Microscopy

The surface morphology of the modified wheat straw and the superabsorbent polymer (SAP) samples was visualized using a JEOL JSM-6380LV scanning electron microscope (JEOL Ltd., Tokyo, Japan). The microscope was operated in secondary electron imaging (SEI) mode to capture the images. To ensure sufficient electrical conductivity, all samples were sputter-coated with a 10 nm gold film before examination.

#### 4.4.3. X-Ray Diffraction

The crystallinity of the modified wheat straw samples was evaluated by X-ray diffraction (XRD) using an Empyrean diffractometer (Malvern Panalytical B.V., Almelo, The Netherlands). Data were collected with Cu-Kα radiation (λ = 1.54 Å) at 45 kV and 35 mA, scanning the 2θ range from 10° to 80° with a step size of 0.02° and a speed of 2°/min.

The crystallinity index (*CrI*) was calculated from the XRD data using the Segal peak height method [52]:

(3) $$CrI=\;\frac{I_{max}-\;I_{AM}}{I_{max}}\times 100,$$

where *I_max_* is the intensity of the most intense peak between 2θ = 20–23°, and *I_AM_* is the intensity of the amorphous peak at 2θ = 18°.

#### 4.4.4. Thermogravimetric Analysis and Differential Scanning Calorimetry

The structural properties of the SAP samples were verified using thermogravimetric analysis (TGA) and differential scanning calorimetry (DSC). The experiments were conducted on a Netzsch STA 449 F3 Jupiter simultaneous thermal analyzer under a helium atmosphere. Samples of approximately 7 mg were heated in sealed aluminum pans from 30 °C to 600 °C at a rate of 10 °C/min.

#### 4.4.5. Rheological Studies

Rheological properties of the SAPs were evaluated using an MCR102 (Anton Paar, Graz, Austria) rotational rheometer with a “plate–plate” measuring system (two 25 mm diameter plates with a gap 1 mm). The temperature control of samples was fulfilled with the lower heating system and active casing both using the Peltier elements P-PTD200 (Anton Paar, Graz, Austria). The variation in given temperature was within ±0.1 °C. Measurements were carried out in the following deformation modes: periodic oscillations at constant temperature (25 °C) with different amplitudes (γ = 0.04–58%) at a constant frequency ω = 6.28 rad/s, or varying frequency (ω = 0.1–300 rad/s) at constant amplitude γ = 0.1%.

### 4.5. Investigating of the SAP Swelling Performance

The equilibrium swelling ratio and swelling kinetics in distilled water and a 0.15 M NaCl aqueous solution were determined according to the method described in [40]. Briefly, SAP samples (0.2000 ± 0.0002 g) were immersed for 24 h in either 500 mL of distilled water or 100 mL of 0.15 M NaCl solution (pH = 6.6 ± 0.2; 25 ± 2 °C) to reach swelling equilibrium. The swollen samples were then collected by filtration and gently blotted dry with filter paper. The equilibrium swelling ratio, *Q_e_*, and the swelling ratio at time *t*, *Q_t_*, are calculated as

(4) $$Q=\frac{m_{1}-m_{0}}{m_{0}},$$

where *m*_1_ and *m*_0_ are the weights of the swollen and dry SAP samples, respectively. Three samples were tested for each synthesized SAP (*n* = 3, *p* = 0.95). The results are presented as mean values ± standard deviation based on three independent experiments, analyzed using MS Excel software.

To elucidate the water absorption mechanism, the swelling kinetics data were analyzed by fitting to established kinetic models commonly applied to hydrogel systems [53,54,55]. The pseudo-first-order swelling kinetic model is represented as [56]:

(5) $$\ln\left(Q_{e}-\;Q_{t}\right)=\ln Q_{e}-\;k_{1}t\;,$$

the pseudo-second-order swelling kinetic model is described by the following equations [57]:

(6) $$\frac{t}{Q_{t}}=\;\frac{1}{k_{2}Q_{e}^{2}}+\;\frac{t}{Q_{e}},$$

and the Ritger–Peppas model which is valid for the initial 60% of the equilibrium swelling ratio and characterized as [58]:

(7) $$F=\;\frac{Q_{t}}{Q_{e}}=k\times t^{n},$$

or the logarithm Expression (8):

(8) $$\ln F=\ln Q_{t}-\ln Q_{e}=\ln k+n\ln t,$$

where *Q_e_* and *Q_t_* represent the swelling ratios at equilibrium and at time *t*, respectively; *k*_1_ (1/min) and *k*_2_ (g/(mg min)) are the rate constants for the pseudo-first-order and pseudo-second-order models, respectively; *F* denotes the fractional swelling rate at time *t*; *k* is the structural parameter; and *n* is the swelling exponent indicating the type of diffusion mechanism. The applicability of each model was evaluated based on the coefficient of determination (R^2^).

For determining the diffusion coefficient, a short-time approximation method was applied [59], which is valid for the initial 60% of the equilibrium swelling ratio. For spherical SAP particles, the diffusion coefficient, *D*, is expressed by the following equation [59]:

(9) $$\frac{Q_{t}}{Q_{e}}=4\sqrt{\frac{Dt}{\pi r^{2}}},$$

where *D* (cm^2^/min) is the diffusion coefficient, *t* (min) is time, and *r* (cm) is particle radius. The diffusion coefficient was determined from the slope *Q_t_*/*Q_e_* versus *t*^1/2^ by equation:

(10) $$k_{D}=4\sqrt{\frac{D}{\pi r^{2}}}.$$

### 4.6. Nitrogen Fertilizer Retention

Quartz sand, pre-washed with distilled water and dried at 300 °C to constant weight, was mixed with the SAP (particle size < 1.0 mm) in a 1:10 volume ratio. The resulting mixture was packed into a glass tube (100 mm in length, 10 mm in diameter) sealed at one end with a nylon cloth. Then, 25 mL of a 92 mM aqueous urea solution was passed through the tube at a flow rate of 10 mL/min.

The volume of the eluted solution was measured, and the urea concentration was determined according to the method described in [60]. Sand without the added SAP was used as a control sample.

Data are presented as the mean ± standard deviation from three independent experiments (*n* = 3, *p* = 0.95) and were processed using MS Excel.

## Figures and Tables

**Table 1:** Equilibrium swelling ratio values for the synthesized SAPs.

SAPs	Equilibrium Swelling Ratio, *Q_e_*, g/g	
	Distilled Water	0.15 M NaCl
SAP-WS-20	376 ± 5	38 ± 1
SAP-WS-33	203 ± 6	34 ± 1
SAP-CMWS-Ac-20	515 ± 7	44 ± 1
SAP-CMWS-Ac-33	426 ± 8	40 ± 1
SAP-CMWS-Al-20	566 ± 12	50 ± 2
SAP-CMWS-Al-33	486 ± 17	46 ± 2
Aquasorb^®^	474 ± 29	55 ± 5
Akvasin^®^	459 ± 40	53 ± 4

**Table 2:** Parameters of the swelling kinetics models and the calculated diffusion coefficients.

SAPs	Pseudo-First Order		Pseudo-Second Order		Ritger–Peppas Model		*D*·10^10^, cm^2^/min
	R^2^	*k*_1_, 1/min	R^2^	*k*_2_, g/(mg min)	R^2^	*n*	
SAP-WS-20	0.98	0.008	0.99	0.002	0.99	0.68	0.221 ± 0.02
SAP-WS-33	0.98	0.007	0.99	0.004	0.99	0.68	0.242 ± 0.01
SAP-CMWS-Ac-20	0.95	0.010	0.99	0.002	0.99	0.73	0.241 ± 0.01
SAP-CMWS-Ac-33	0.97	0.006	0.99	0.002	0.99	0.88	0.324 ± 0.02
SAP-CMWS-Al-20	0.98	0.012	0.99	0.002	0.99	0.83	0.324 ± 0.01
SAP-CMWS-Al-33	0.88	0.007	0.99	0.002	0.99	0.87	0.338 ± 0.01

**Table 3:** Urea retention quantification *.

SAP	*C*_0_, mM	*C*_1_, mM	*V*_0_, mL	*V*_1_, mL	Sorbed urea, %
Control (sand without SAP)	92 ± 1	90 ± 0.8	25	22.0 ± 1.5	13.9 ± 1.5
SAP-CMWS-Ac-20		86 ± 1.3		18.5 ± 1.0	30.8 ± 2.4
SAP-CMWS-Al-20		80 ± 0.7		18.5 ± 1.1	35.7 ± 2.0
SAP-CMWS-Ac-33		82 ± 1.1		17.0 ± 0.9	39.4 ± 2.3
SAP-CMWS-Al-33		81 ± 0.6		12.5 ± 0.7	56.0 ± 2.5

* *C*_0_ and *C*_1_ are the urea concentrations (mM), and *V*_0_ and *V*_1_ are the solution volumes (mL), before and after column permeation, respectively. Sorbed urea, % is the percentage of urea (from the total amount passed through the column) that was sorbed by the SAP.

**Table 4:** The SAP synthesis conditions.

WS or WS-Derived Feed, g	WS or WS-Derived Feed in SAP, wt. %	AAm, g	AA *, g	MBAAm, g	MBAAm: Acrylate Comonomers Ratio (*w*/*w*)	PPS, g
0.25	20%	0.25	0.80	0.003	1:350	0.015
0.50	33%				1:350	

* Prior to synthesis, AA was subjected to 70% mol. neutralization using KOH.

## Data Availability

The original contributions presented in the study are included in the article/Supplementary Materials, and further inquiries can be directed to the corresponding author.

## Supplementary Materials

The following supporting information can be downloaded at: https://www.mdpi.com/article/10.3390/gels11120953/s1, Figure S1: Swelling data analysis using for SAP-20 series processed by: pseudo-first-order model (a); pseudo second-order model (b); Ritger–Peppas model (c); diffusion coefficient evaluation (d); Figure S2: Swelling data analysis using for SAP-33 series processed by: pseudo-first-order model (a); pseudo second-order model (b); Ritger–Peppas model (c); diffusion coefficient evaluation (d).
