# Supplementary material for: Transforming Wheat Straw into Superabsorbent Polymers for Sustainable Agricultural Management
Source: Gels. 2025 Nov 27;11(12):953. doi: 10.3390/gels11120953 (PMC12732788; doi:10.3390/gels11120953)
Supplement: Supplementary file 1 [file gels-11-00953-s001.zip › gels-3990334-supplementary.pdf]

Transforming Wheat Straw into Superabsorbent Polymers for Sustainable Agricultural Management

Andrey V. Sorokin, Aidar I. Kadyirov, Igor A. Saranov, Egor M. Tsimmer, Vladislav A. Kiselev, Ivan A. Zhuravlev and Maria S. Lavlinskaya

Supplementary Materials

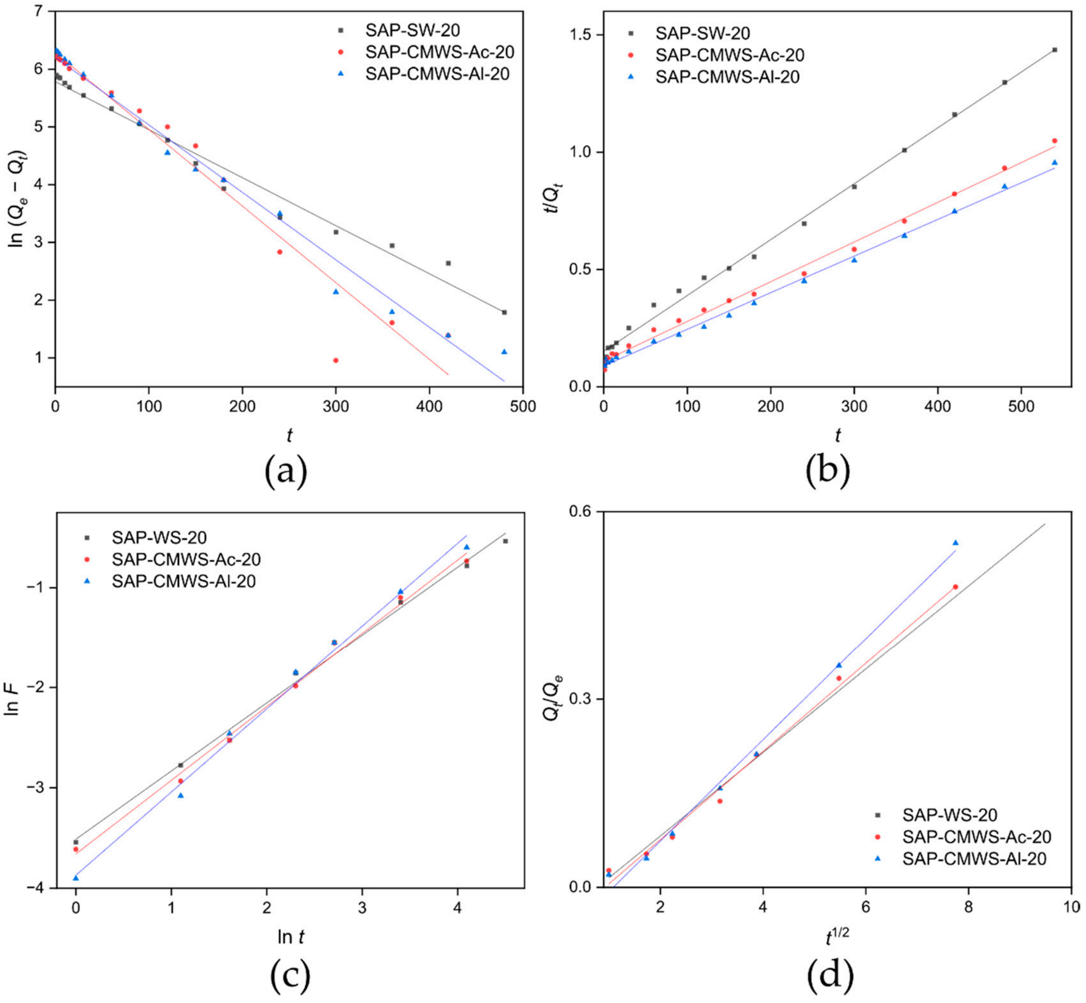

Figure S1. Swelling data analysis using for SAP-20 series processed by: pseudo-first-order model **(a)**; pseudo second-order model **(b)**; Ritger-Peppas model **(c)**; diffusion coefficient evaluation **(d)**.

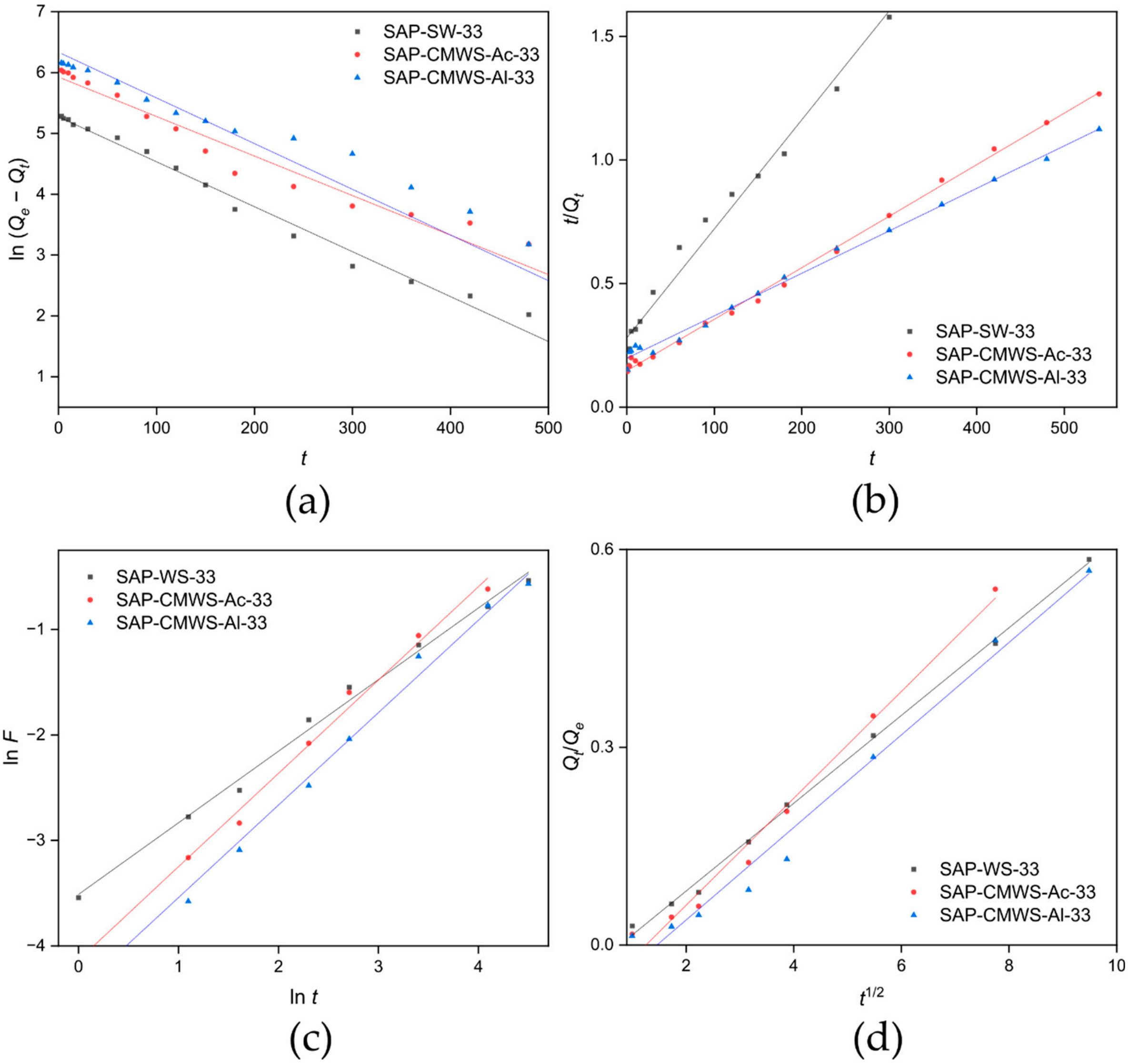

Figure S2. Swelling data analysis using for SAP-33 series processed by: pseudo-first-order model **(a)**; pseudo second-order model **(b)**; Ritger-Peppas model **(c)**; diffusion coefficient evaluation **(d)**.
